# Enhanced diagnostic accuracy of high‐grade cervical intraepithelial neoplasia in postmenopausal women through *PAX1* / *JAM3* methylation analysis

**DOI:** 10.1002/ijc.70245

**Published:** 2025-11-14

**Authors:** Huanzi Peng, Jing Li, Qun Zhou, Hui Zhou, Jiaqi Peng, Jing Wang, Pei Liu, Kun He, Wene Liu, Ping Tan, Lei Li, Xiaobing Xie

**Affiliations:** ^1^ Medical Laboratory Center The First Hospital of Hunan University of Chinese Medicine Changsha China; ^2^ Medical Laboratory Center QingHai Red Cross Hospital Qinghai China; ^3^ Department of Medical Laboratory Beijing Origin‐Poly Bio‐Tec Co., Ltd Beijing China; ^4^ Department of Obstetrics and Gynecology Peking Union Medical College Hospital Beijing China

**Keywords:** colposcopy, *PAX1*/*JAM3* methylation, postmenopausal womencervical cancer screening

## Abstract

This study aimed to assess the diagnostic‐support and triage value of PAX1/JAM3 methylation testing for identifying high‐grade cervical lesions among postmenopausal women referred for colposcopy. A total of 216 women aged ≥50 years who underwent colposcopy due to positive high‐risk human papillomavirus (hrHPV)/cytology detection and/or abnormal clinical symptoms were included, and 212 women aged <50 years matched 1:1 by hrHPV and cytology results as the control group. The effectiveness of *PAX1*/*JAM3* methylation in detecting high‐grade cervical intraepithelial neoplasia (CIN) was compared to traditional screening methods. *PAX1/JAM3* methylation showed a sensitivity of 93.2%[85.7%–100%] for detecting CIN2+ lesions (97.2%[91.9%–100%] for CIN3+), with a specificity of 93.6%[89.9%–97.3%] for CIN1‐lesions, outperforming liquid‐based cytology (LBC) (CIN2+/CIN3+ sensitivity: 75%[60.9%–89.1%]; specificity: 52.3%[44.9%–59.8%]) and the combination of LBC and hrHPV tests according to current guidelines (CIN2+/CIN3+ sensitivity: 81.8%[70.4%–93.2%]/83.3%[71.2%–95.5%]; specificity: 45.3%[37.9%–52.8%]). Methylation detection successfully identified two adenocarcinoma cases with negative hrHPV and LBC, as well as 7 patients with non‐16/18 hrHPV infection that were missed by LBC. The methylation levels of *PAX1* and *JAM3* were elevated in elderly women with CIN2+ compared to younger women, and showed no association with HPV types. In conclusion, *PAX1/JAM3* methylation testing showed promising diagnostic‐support performance among postmenopausal women referred for colposcopy, suggesting potential utility as a supplementary detection method to improve diagnostic accuracy in older women.

## Graphical abstract

What's new?

Cervical cancer screening is challenging in postmenopausal women, partly due to physiological changes that reduce the sensitivity of cytology and colposcopy. This study evaluated the diagnostic performance of combined *PAX1* and *JAM3* gene methylation testing in a large cohort of postmenopausal women undergoing colposcopy. Compared to cytology and high‐risk HPV‐based screening strategies, *PAX1/JAM3* methylation offered higher specificity and comparable or improved sensitivity. The test identified cervical adenocarcinomas and high‐grade lesions missed by standard methods, particularly in women with non‐16/18 HPV infection or normal cytology. Methylation testing holds potential clinical value in enhancing cervical cancer diagnosis among women aged over 50.

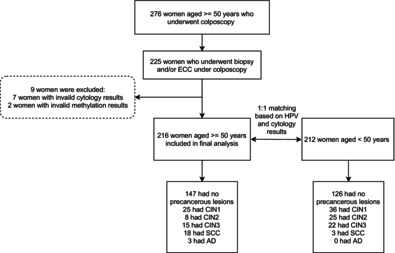

**Abbreviations**
AD: adenocarcinoma
ASC‐H: atypical squamous cells, cannot exclude HSIL
ASC‐US: atypical squamous cells of undetermined significance
AUC: area under the curve
CIN: cervical intraepithelial neoplasia
CISCER: PAX1/JAM3 methylation test
Ct–: cycle threshold
ECC: endocervical curettage
HPV: human papillomavirus
hrHPV: high‐risk human papillomavirus
HSIL: high‐grade squamous intraepithelial lesion
JAM3: junctional adhesion molecule 3
LBC: liquid‐based cytology
LSIL: low‐grade squamous intraepithelial lesion
NILM: negative for intraepithelial lesion or malignancy
PAX1: paired box gene 1
SCC: squamous cell carcinoma
SCJ: squamocolumnar junction
TZ3: transformation zone type 3

## 1 INTRODUCTION

Cervical cancer (CC) is the fourth most common cancer in terms of both incidence and mortality in women, with an estimated 660,000 new cases and 350,000 deaths worldwide in 2022.^1^ Persistent infection with human papillomavirus (HPV) is widely acknowledged as a significant risk factor for the development of cervical cancer.^2^, ^3^ While the incidence of CC has decreased in developed countries over the past few decades due to policies, it continues to be a leading cause of both incidence and mortality among women in developing countries and specific populations.^4^, ^5^ CC remains a serious public health issue in many low‐income and middle‐income regions. In China, the incidence and mortality rates of CC continue to increase in 2022, with 150,700 new cases and 37,200 deaths reported.^6^

The incidence and mortality rates of CC among young women in China have shown a decrease, particularly notable in urban areas. However, the age‐standardized incidence rate and mortality rate for women over 50 years old have exhibited an increase in both urban and rural areas.^7^, ^8^ Women aged over 50, particularly postmenopausal women, may experience cervical atrophy as a result of hormonal downregulation. This can result in the migration of the area [HPV infection or cervical intraepithelial neoplasia (CIN) occurrence] into the cervical canal, which is known as Transformation Zone 3 (TZ3).^9^, ^10^ Due to various reasons, postmenopausal women may face some specific difficulties in CC screening and diagnosis, including physiological changes, increased false negative rate, insufficient screening awareness, unobvious symptoms, and comorbidities. This physiological alteration reduces the sensitivity of cytology and colposcopy in detecting CIN2 and severe lesions (CIN2+), complicating clinical management and presenting challenges to CC screening strategies.^11^, ^12^, ^13^, ^14^ As age increases, the therapeutic efficacy and prognosis of CC decline. It has been reported that 9.91% of elderly women delay treatment due to late diagnosis.^12^ Hence, it is important to develop more precise methods for detecting CIN2+ in elderly women to ensure the overall effectiveness of diagnosis and treatment.

Numerous studies have extensively summarized a significant relationship between oncogenesis and epigenetic mediators, including histone modifications, protein and non‐coding RNA interactions, nucleosome occupancy and positioning, and direct DNA modifications.^15^, ^16^, ^17^ In general, the increased methylation of CPG‐rich islands in the promoters of human tumor suppressor genes can lead to the silencing of gene transcription, thereby initiating the development of cancer. This theory has become the basis for methylation detection in cervical cancer.^16^, ^18^, ^19^, ^20^

The junctional adhesion molecule‐3 (*JAM3*) plays a crucial role in leukocyte migration, angiogenesis, and tumor metastasis.^21^ Literature showed a higher sensitivity of *JAM3* methylation testing in high risk‐HPV (hrHPV)‐positive women compared to liquid‐based cytology (LBC).^22^, ^23^ The paired box gene 1 (*PAX1*), a gene transcription factor that is highly conserved across both vertebrates and invertebrates, plays a crucial role in various biological processes. Numerous studies have recognized *PAX1* as a methylated silencing gene observed in cervical cancer. The methylated *PAX1* gene is a potential biomarker to enhance the effectiveness of CC screening.^24^, ^25^, ^26^ The combination of *PAX1* and *JAM3* methylation detection has been reported to be significantly higher in cases of HPV persisting infection for more than 3 years compared to those with less than 3 years, and it had high performance in detecting CIN2+ for women with hrHPV infection.^27^, ^28^, ^29^, ^30^, ^31^

This study focused on women aged 50 and above, aiming to evaluate the clinical value of *PAX1* and *JAM3* methylation testing in cervical exfoliated cells. We also included test results from women under 50 years of age as a control group to compare variations in the clinical performance of different tests. This study offers scientific evidence to support personalized testing, which could ultimately enhance the optimization of CC prevention strategies and public health policies tailored to this specific population.

## 2 MATERIALS AND METHODS

### 2.1 Study design and participants

From June 2023 to January 2025, patients over 50 years of age who underwent colposcopy due to positive screening results and/or abnormal clinical symptoms at The First Hospital of Hunan University of Chinese Medicine were enrolled. The inclusion criteria were as follows: participants who were postmenopausal, had undergone cytology and hrHPV detection within the past month, agreed to participate in the study, and signed the informed consent form. Participants with a documented history of reproductive tract malignancy or other tumors, or those with known autoimmune disease or currently taking immunosuppressants, were excluded. Additionally, non‐pregnant women under 50 years of age were included for an exploratory analysis, with a 1:1 match based on the HPV and cytology results of women over 50.

### 2.2 Collection of cervical scraping cell specimens and clinical information

After exposing the participant's cervix, delicately cleanse the cervical surface using a sterile cotton swab to remove any facial secretions. Next, use Rovers Cervex‐Brush (Rovers Medical Devices, Oss, The Netherlands) to gently rotate the brush on the cervical surface and uterus in a clockwise motion for 5 to 10 rotations to collect cervical exfoliated cells. Then, place the brush head in PreservCyt® cytology medium (Hologic Inc., MA, USA) and affix the test barcode. The cell specimen was stored in PreservCyt® cytology medium at 4°C.

The basic information of participants was gathered, encompassing general demographics, LBC and hrHPV DNA test results (specifically HPV16/18 or other types), as well as pathology reports. These records were meticulously documented and reviewed by two research associates.

### 2.3 Cytology, HPV genotyping, and pathology

An 8 mL cell sample solution was prepared and stained using the ThinPrep cytology system (Hologic Inc., MA, USA). The cell slides were diagnosed by two pathologists in accordance with the Bethesda 2014 classification criteria (TBS).^32^ The TBS defines cytological morphology as follows: negative for intraepithelial lesion or malignancy (NILM), atypical squamous cells of undetermined significance (ASC‐US), atypical glandular cells, low‐grade squamous intraepithelial lesion (LSIL), atypical squamous cells cannot exclude high‐grade squamous intraepithelial lesion (ASC‐H), high‐grade squamous intraepithelial lesion (HSIL), squamous cell carcinoma (SCC), as well as adenocarcinoma (AD).

hrHPV detection was conducted using the Cobas 4800 HPV Controls Kit (Roche, Shanghai, China) following the manufacturer's instructions. The test results provided the detection of HPV types 16 and 18, as well as 12 other high‐risk HPV types, collectively referred to as non‐16/18 hrHPV: HPV 31/33/35/39/45/51/52/56/58/59/66/68.

All participants underwent colposcopy examination, and colposcopy‐directed biopsies were performed on visible lesions or 1–2 random biopsies were taken from the normal‐appearing cervix, unless the patient refused to undergo a biopsy. For women with TZ3, endocervical curettage (ECC) was performed. Two senior pathologists independently evaluated the pathological biopsy results. In cases of discrepancy between the two pathologists, a third pathologist was consulted to reach a consensus diagnosis. Patients with pathological results indicating the need for surgical intervention underwent a loop electrosurgical excision procedure, cold knife conization, or total hysterectomy. The final diagnostic results (end‐point) were categorized as cervicitis (No CIN), CIN1, CIN2, CIN3, or CC.

### 2.4 *PAX1* and *JAM3* methylation detection

Genomic DNA (gDNA) was extracted from the remaining cervical samples preserved in PreservCyt® solution using the JH‐DNA Isolation and Purification kit (OriginPoly Bio‐Tec Co., Ltd., Beijing, China) according to the manufacturer's instructions. The quantity and quality of DNA were assessed using a NanoDrop 2000c spectrophotometer (Thermo Fisher Scientific, DE, USA). Briefly, 200–1000 ng of gDNA were subjected to bisulfite conversion using JH‐DNA Methylation‐Lightning MagPrep (OriginPoly Bio‐Tec Co., Ltd., Beijing, China). The methylation levels of *PAX1* (*PAX1* ^*m*^) and *JAM3* (*JAM3* ^*m*^) were determined using the CISCER® DNA Methylation Detection Kit (OriginPoly Bio‐Tec Co., Ltd., Beijing, China)—approved by the China National Medical Products Administration (NMPA) as a Class III medical device (No. 20233400253)—with the SLAN‐96S real‐time PCR System (Shanghai Hongshi Medical Technology Co., Ltd., China), in accordance with the manufacturer's instructions. The real‐time PCR procedure was performed as follows: initial denaturation at 96°C for 10 min (1 cycle), followed by 45 cycles of denaturation at 94°C for 15 s, annealing at 64°C for 5 s, and extension at 60°C for 30 s. The reaction was concluded with a final cooling step at 25°C for 1 min (1 cycle). The methylation status of *PAX1* and *JAM3* genes was determined by calculating the difference between their respective Ct values and that of *GAPDH* (used as an internal control), expressed as Δ*CtP* = *CtPAX1* − Ct*GAPDH* for *PAX1* and Δ*CtJ* = *CtJAM3* − Ct*GAPDH* for *JAM3*, with a positive result for the CISCER (*PAX1* ^*m*^/*JAM3* ^*m*^) test. The pre‐established Δ*Ct* cut‐off values (P‐type ≤6.6, J‐type ≤10.0) were clinically validated in accordance with the standards set forth by the National Medical Products Administration (NMPA) of China and received approval as part of the registration process for Class III medical devices. The classification of results as positive or negative in this study is based on the setting values provided in the package insert of the kit. Comparable cutoffs and performance characteristics have been reported in independent studies that used the same kit or validated the same.^29^, ^31^

### 2.5 Data and statistical analysis

The statistical analyses in this study were comprehensively executed utilizing R version 4.2.1 (released on 2022‐06‐23). The generation of receiver operating characteristic curves, along with their respective areas under the curve and 95% confidence intervals, relied on the pROC package, version 1.18.0. Moreover, the sensitivity, specificity, positive predictive value (PPV), and negative predictive value (NPV), alongside their 95% confidence intervals, were accurately calculated using the epiR package (version 2.0.38) and the bdpv package (version 1.3). Categorical variables were presented as percentages of total counts, while continuous variables were expressed as medians accompanied by interquartile ranges (Q1–Q3) or as mean values with their standard deviations. To compare continuous variables between groups, the Wilcoxon rank‐sum test was employed, while categorical variables were analyzed using the chi‐square test or Fisher's exact test. For all statistical tests, a *p*‐value of 0.05 or less was deemed significant.

## 3 RESULTS

### 3.1 Participant characteristics

**FIGURE 1:**
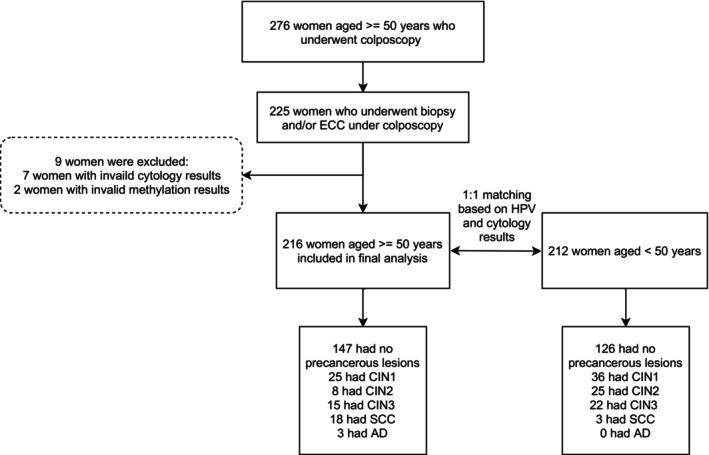
Flowchart of this study. AD, adenocarcinoma; CIN, cervical intraepithelial neoplasia; ECC, endocervical curettage; SCC, squamous cell carcinoma.

This study enrolled 276 women aged 50 years or older, all of whom underwent colposcopy due to positive screening results and/or abnormal clinical symptoms. The further analysis was conducted on 216 women who underwent cervical biopsy and/or ECC, and had available results from both cytological and hrHPV genotyping tests. Additionally, 212 women under the age of 50 were included for exploratory analysis (Figure 1, Table 1).

**TABLE 1:** Characteristics of participants.

	Overall (*N* = 428)	< 50 years (*N* = 212)	> = 50 years (*N* = 216)	*p*‐value
Age Median [Q1, Q3]	50.0 [36.0,57.0]	36.0 [31.8,41.0]	57.0 [53.0,62.0]	
Pathology				<.001
No CIN	273 (63.8%)	126 (59.4%)	147 (68.1%)	
CIN1	61 (14.3%)	36 (17.0%)	25 (11.6%)	
CIN2	33 (7.7%)	25 (11.8%)	8 (3.7%)	
CIN3	37 (8.6%)	22 (10.4%)	15 (6.9%)	
Squamous cell carcinoma	21 (4.9%)	3 (1.4%)	18 (8.3%)	
Adenocarcinoma	3 (0.7%)	0 (0%)	3 (1.4%)	
HPV genotyping				.8
Negative	76 (17.8%)	39 (18.4%)	37 (17.1%)	
HPV 16/18	107 (25.0%)	50 (23.6%)	57 (26.4%)	
Non‐16/18 hrHPV	245 (57.2%)	123 (58.0%)	122 (56.5%)	
Cytology results				>.9
NILM	204 (47.7%)	103 (48.6%)	101 (46.8%)	
ASC‐US	109 (25.5%)	55 (25.9%)	54 (25.0%)	
LSIL	56 (13.1%)	28 (13.2%)	28 (13.0%)	
ASC‐H	22 (5.1%)	10 (4.7%)	12 (5.6%)	
HSIL	31 (7.2%)	14 (6.6%)	17 (7.9%)	
Cervical cancer	6 (1.4%)	2 (0.9%)	4 (1.9%)	
Methylation results				
Δ*CtPAX1*	13.7 [8.23, 16.9]	12.2 [8.47, 16.9]	15.2 [7.82, 16.9]	>.9
Δ*CtJAM3*	15.1 [12.7, 16.8]	14.8 [12.7, 16.8]	15.4 [12.6, 16.8]	>.9

Abbreviations: ASC‐US, atypical squamous cells of undetermined significance; ASC‐H, atypical squamous cells, cannot exclude HSIL; CIN, cervical intraepithelial neoplasia; hrHPV, high‐risk human papillomavirus; HPV‐16/18, HPV16 and (or) HPV18 types; Non‐16/18 hrHPV, HPV 31, 33, 35, 39, 45, 51, 52, 56, 58, 59, 66 and 68; HSIL, high‐grade squamous intraepithelial lesion; LSIL, low‐grade squamous intraepithelial lesion; no CIN, no CIN found; NILM, no intraepithelial lesions or malignancy.

Among the 216 women aged ≥50, 179 (82.9%) tested positive for hrHPV, including 57 (26.4%) positive for HPV 16/18 and 122 (56.5%) positive for non‐HPV16/18 types. The cytological results for these women were as follows: 101 (46.8%) had NILM, 54 (25.0%) had ASC‐US, 28 (13.0%) had LSIL, 12 (5.6%) had ASC‐H, 17 (7.9%) had HSIL, and 4 (1.9%) had cancer. Pathological findings revealed that 147 women (68.1%) had no CIN, 25 (11.6%) had CIN1, 8 (3.7%) had CIN2, 15 (6.9%) had CIN3, 18 (8.3%) had squamous cell carcinoma (SCC), and 3 (1.4%) had AD.

Compared to the elderly women with consistent cytological and HPV results, women under 50 years of age exhibited higher proportions of CIN1 (17.0% vs. 11.6%), CIN2 (11.8% vs. 3.7%), and CIN3 (10.4% vs. 6.9%), and lower proportions of cancer cases (1.4% vs. 9.7%).

### 3.2 *PAX1* and *JAM3* methylation in varying grades of cervical lesions and different HPV types

**FIGURE 2:**
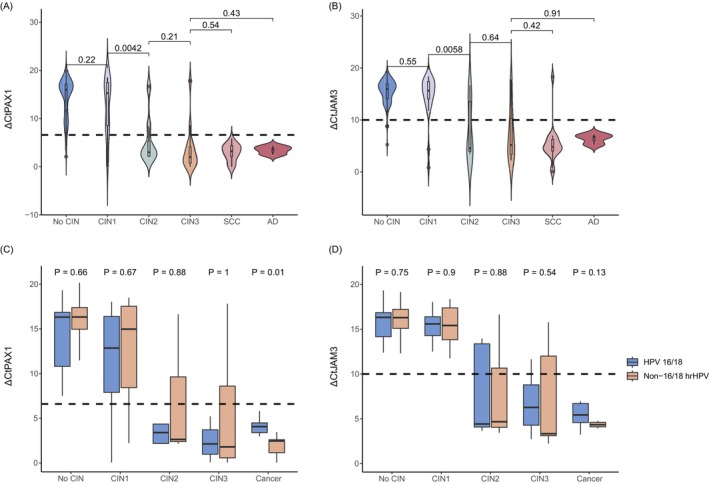
The methylation of *PAX1* and *JAM3* gene in women aged ≥50 with different grades of cervical lesions. (A) Δ*Ct* values of *PAX1* gene in different grade of lesions. (B) Δ*Ct* values of *JAM3* gene in different grade of lesions. (C) Δ*Ct* values of *PAX1* gene in patients who infected with HPV 16/18 or non‐16/18 hrHPV. (D) Δ*Ct* values of *JAM3* gene in patients who infected with HPV 16/18 or non‐16/18 hrHPV. AD, adenocarcinoma; CIN, cervical intraepithelial neoplasia; SCC, squamous cell carcinoma; HPV16/18: HPV16 and (or) HPV18 types; Non‐16/18 hrHPV: HPV 31, 33, 35, 39, 45, 51, 52, 56, 58, 59, 66 and 68.

The methylation levels of *PAX1* and *JAM3* genes in women aged over 50 years with different grades of cervical lesions were shown in Figure 2. For both genes, Δ*Ct* values were higher in women without CIN and those with CIN1, with no significant difference between the two groups (Figure 2A,B). In contrast, a marked decrease in Δ*Ct* values was observed in the CIN2+ groups, with values progressively decreasing as lesion severity increased. No significant differences were found in the methylation levels of *PAX1* and *JAM3* between elderly women infected with HPV16/18 or non‐HPV16/18 high‐risk HPV types (Figure 2C,D). This suggests that methylation levels are not influenced by the HPV type, only linked to disease severity.

Compared to elderly women, women under 50 years of age exhibited a similar trend in the methylation expression of *PAX1* and *JAM3* across different lesion grades. Notably, younger women showed higher Δ*Ct* values in CIN2+ lesions, particularly for the *PAX1* gene in the HPV16/18‐infected group (Supp. Figure 1).

### 3.3 Effectiveness of different screening tests

For the detection of CIN2+ lesions in women aged ≥50, hrHPV DNA detection showed high sensitivity at 95.5% (95% CI: 89.3%–100%), but low specificity at 20.3% (95% CI: 14.3%–26.4%), with an AUC of 0.579 (95% CI: 0.518–0.64). When considering only HPV 16/18 infections, the sensitivity dropped to 61.4% (95% CI: 47%–75.8%). In contrast, the sensitivities of *PAX1* ^*m*^ (90.9% [95% CI: 82.4%–99.4%], *p* = 1), *JAM3* ^*m*^ (81.8% [95% CI: 70.4%–93.2%], *p* = .676), and their combination (93.2% [95% CI: 85.7%–100%], *p* = .089) were slightly lower than that of hrHPV detection (*p* = 1), yet these markers exhibited much higher specificities: 94.8% (95% CI: 91.4%–98.1%, *p* = 1.20e‐43), 97.1% (95% CI: 94.6%–99.6%, *p* = 1.21e‐54), and 93.6% (95% CI: 89.9%–97.3%, *p* = 3.39e‐42), respectively. Their AUCs were 0.928 (95% CI: 0.869–0.987), 0.895 (95% CI: 0.825–0.964), and 0.934 (95% CI: 0.878–0.989). Furthermore, the performance of methylation testing was significantly superior to that of cytological testing (CISCER vs. LBC≥ASC‐US: *P*_sensitivity_ = 0.039, *P*_specificity_ = 1.93e‐17), and even outperformed the combination of cytology and hrHPV detection (CISCER vs. LBC&hrHPV: *P*_sensitivity_ = 0.196, *P*_specificity_ = 7.95e‐22) (LBC and hrHPV was defined as positive in cases of HPV 16/18 infection, non‐16/18 hrHPV infection with cytology ≥ ASC‐US, or HPV‐negative with cytology > ASC‐US, while all other cases were considered negative). Similar findings were observed in the detection of CIN3+ lesions, where the higher sensitivity of CISCER compared to the hrHPV test was attributed to the fact that 2 adenocarcinoma patients were hrHPV‐negative (Table 2, Supp. Table 1).

**TABLE 2:** Clinical performance of different tests in triaging women over 50 years.

	Sensitivity % (95% CI)	Specificity % (95% CI)	PPV (95% CI)	NPV % (95% CI)	AUC
CIN2+ lesions					
*PAX1* ^*m*^	90.9 [82.4–99.4]	94.8 [91.4–98.1]	81.6 [70.8–92.5]	97.6 [95.3–99.9]	0.928 [0.869–0.987]
*JAM3* ^*m*^	81.8 [70.4–93.2]	97.1 [94.6–99.6]	87.8 [77.8–97.8]	95.4 [92.3–98.5]	0.895 [0.825–0.964]
CISCER	93.2 [85.7–100]	93.6 [89.9–97.3]	78.8 [67.7–89.9]	98.2 [96.1–100]	0.934 [0.878–0.989]
LBC (≥ASC‐US)	75 [62.2–87.8]	52.3 [44.9–59.8]	28.7 [20.4–37]	89.1 [83–95.2]	0.637 [0.535–0.738]
hrHPV+	95.5 [89.3–100]	20.3 [14.3–26.4]	23.5 [17.3–29.7]	94.6 [87.3–100]	0.579 [0.518–0.64]
HPV16/18+	61.4 [47–75.8]	82.6 [76.9–88.2]	47.4 [34.4–60.3]	89.3 [84.5–94.1]	0.72 [0.619–0.82]
LBC and hrHPV	81.8 [70.4–93.2]	45.3 [37.9–52.8]	27.7 [20–35.4]	90.7 [84.6–96.8]	0.636 [0.542–0.73]
CIN3+ lesions					
*PAX1* ^*m*^	94.4 [87–100]	91.7 [87.6–95.7]	69.4 [56.5–82.3]	98.8 [97.2–100.5]	0.931 [0.873–0.988]
*JAM3* ^*m*^	86.1 [74.8–97.4]	94.4 [91.1–97.8]	75.6 [62.5–88.8]	97.1 [94.7–99.6]	0.903 [0.83–0.976]
CISCER	97.2 [91.9–100]	90.6 [86.3–94.8]	67.3 [54.6–80.1]	99.4 [98.2–100.6]	0.939 [0.891–0.987]
LBC (≥ASC‐US)	75 [60.9–89.1]	51.1 [43.8–58.4]	23.5 [15.7–31.2]	91.1 [85.5–96.6]	0.631 [0.523–0.738]
hrHPV+	94.4 [87–100]	19.4 [13.7–25.2]	19 [13.2–24.7]	94.6 [87.3–101.9]	0.569 [0.503–0.636]
HPV16/18+	61.1 [45.2–77]	80.6 [74.8–86.3]	38.6 [26–51.2]	91.2 [86.8–95.6]	0.708 [0.6–0.817]
LBC and hrHPV	83.3 [71.2–95.5]	44.4 [37.2–51.7]	23.1 [15.8–30.3]	93 [87.6–98.4]	0.639 [0.542–0.736]

*Note*: LBC (≥ASC‐US): the positive liquid‐based cytology results were defined as cytology ≥ASC‐US (ASC‐US, LSIL, ASC‐H, HSIL, SCC and AD); hrHPV+: positive in cases infected by HPV 16, 18, 31, 33, 35, 39, 45, 51, 52, 56, 58, 59, 66 or 68; HPV16/18+: positive in cases of HPV 16/18 infection; LBC and hrHPV: positive in cases of HPV 16/18 infection, non‐16/18 hrHPV infection with cytology ≥ ASC‐US, or HPV‐negative with cytology >ASC‐US, while all other cases were considered negative.

Abbreviations: AUC: area under the curve; CI, confidence interval; CISCER: *PAX1* ^*m*^/*JAM3* ^*m*^; *JAM3* ^*m*^, the methylation of *JAM3* gene; NPV, negative predictive value; PPV, positive predictive value; *PAX1* ^*m*^, the methylation of *PAX1* gene.

In women under 50, cytological testing exhibited higher sensitivity than in older women, whereas methylation testing showed the opposite trend. CISCER demonstrated a higher specificity of 94.4% (95% CI: 90.9%–98%) for CIN1‐lesions, while its sensitivity for CIN2+ lesions was lower than traditional methods. This aligns with the lower methylation levels observed in younger women for CIN2 and CIN3 lesions, as shown in Supp. Figure 1, Supp. Table 2.

### 3.4 Significance of methylation‐based triage following traditional screening

**FIGURE 3:**
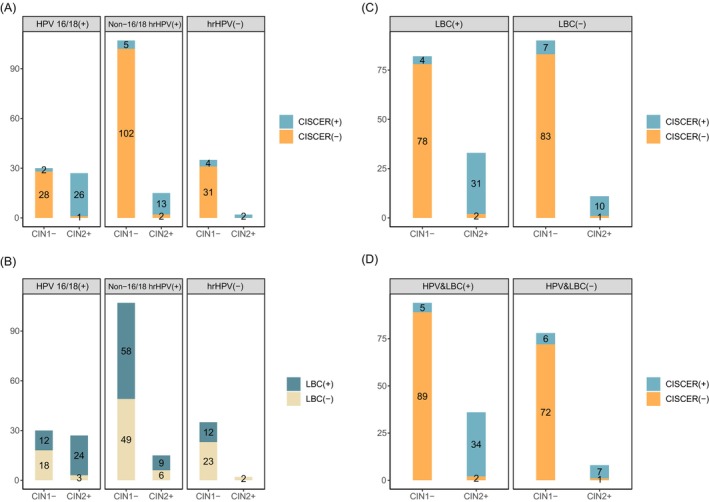
Comparison between *PAX1*/*JAM3* methylation and traditional screening methods in women over 50. (A) Results of *PAX1/JAM3* methylation and hrHPV detection. (B) Results of cytology and hrHPV detection. (C) Results of *PAX1/JAM3* methylation and cytology. (D) Results of *PAX1/JAM3* methylation and the combination of LBC and hrHPV tests. CIN, cervical intraepithelial neoplasia; HPV16/18 (+): HPV16 and (or) HPV18 types; Non‐16/18 hrHPV(+): HPV 31, 33, 35, 39, 45, 51, 52, 56, 58, 59, 66 and 68; CISCER(+): *PAX1* ^*m*^/*JAM3* ^*m*^; LBC(+): Cytology ≥ ASC‐US; hrHPV&LBC(+): Positive in cases of HPV 16/18 infection, non‐16/18 hrHPV infection with cytology ≥ ASC‐US, or HPV‐negative with cytology >ASC‐US, while all other cases were considered negative.

The results indicate that traditional CC screening methods in postmenopausal women may lead to high rates of false positives and false negatives. The incidence of CIN2+ in women infected with HPV 16/18 was significantly higher than in those infected with non‐16/18 hrHPV (47.4% vs. 12.3%, *p* = 6.7e‐7), yet over 50% (30/57) of cases still resulted in false positives. Incorporating CISCER as a triage tool could reduce the false positive rate by more than 90% (HPV16/18: 28/30; non‐16/18 high‐risk HPV: 102/107) (Figure 3A). In contrast, using cytological results for triage would yield much less effective outcomes (HPV16/18: 18/30 vs. 28/30, *p* = .006; non‐16/18 high‐risk HPV: 49/107 vs. 102/107, *p* = 6.23e‐15) and result in more missed diagnoses (HPV16/18: 3/27 vs. 1/27, *p* = .610; non‐16/18 high‐risk HPV: 6/15 vs. 2/15, *p* = .215) (Figure 3B). If HPV type is not considered, CISCER triage would reduce the false positive rate by 95.1% (78/82) and the false negative rate by 90.9% (10/11) in cytological results (Figure 3C).

Notably, two cases of adenocarcinoma in this study, which were negative for hrHPV and had normal cytological results, exhibited high methylation levels of both *PAX1* and *JAM3*. Additionally, 5 cases with non‐16/18 hrHPV‐positive results and NILM, including 2 cases of SCC, 2 cases of CIN3 and one case of CIN2, tested positive for CISCER (Figure 3D). The findings suggest that some cases may be missed after initial screening with traditional HPV testing and even subsequent cytological triage. Methylation tests can effectively reduce the false‐negative rate, particularly in elderly women.

## 4 DISCUSSION

CC is frequently diagnosed in postmenopausal women, yet research focusing on this specific population remains limited. Notably, the HPV infection rate among older women is not lower than that in younger women, and they face an elevated risk of poor prognostic outcomes once diagnosed with cervical cancer.^33^, ^34^ This study further revealed that in cases with similar HPV infection status and cytological results, the incidence of CC in women over 50 was potentially higher than in younger women. These discrepancies may be associated with multiple factors: first, the weakened immune system in older women impairs HPV clearance capacity.^35^, ^36^, ^37^ Second, screening discontinuation or reduced frequency in this age group may delay timely intervention.^38^, ^39^, ^40^ Third, hormonal changes often obscure the squamocolumnar junction (SCJ), reducing the accuracy of colposcopy and increasing diagnostic errors.^12^, ^14^ Collectively, these factors underscore the urgent need for enhanced attention to CC prevention and management in postmenopausal women.

To our knowledge, this study represents the largest sample size to date evaluating methylation markers specifically in postmenopausal women. By assessing the clinical utility of *PAX1/JAM3* methylation detection in cervical exfoliated cells from women over 50, we found a sensitivity of 93.2% [95% CI: 85.7%–100%] and a specificity of 93.6% [95% CI: 89.9%–97.3%] for detecting CIN2+ lesions, performance that surpasses cytology and hrHPV testing.

Previous studies have shown that cellular changes in postmenopausal women reduce the sensitivity of cytology compared to women of reproductive age,^13^, ^41^, ^42^ a finding consistent with the low cytological sensitivity observed in our results. Consequently, relying solely on cytological detection may lead to a higher rate of missed diagnoses. Even when combined with hrHPV testing, current guidelines^40^ may still fail to identify women infected with non‐16/18 hrHPV types. In China, the most prevalent genotypes are HPV 52, 58, 16, and 53,^43^ with non‐HPV 16/18 infections being more common than HPV 16/18. Although non‐HPV 16/18 types have lower carcinogenic potential, they should not be overlooked. Additionally, two cases of adenocarcinoma were identified in this cohort that were neither associated with hrHPV infection nor detected by abnormal cytological results. This observation underscores a critical limitation of HPV testing^40^, ^44^, ^45^: it may miss a subset of malignancies, a gap that cytological testing alone cannot fully address.

In contrast, methylation‐based detection directly measures molecular‐level changes associated with disease progression, independent of HPV genotype or cytological interpretation.^31^ Our results demonstrate that *PAX1/JAM3* methylation testing may significantly mitigate the risk of false‐negative results in CC initial detection, positioning it as a promising triage tool to enhance the accuracy of CC diagnosis. It may serve as a robust supplementary diagnostic approach for postmenopausal women with negative initial screenings or ambiguous colposcopic findings.

We found that the methylation levels of *PAX1* and *JAM3* in elderly women with advanced lesions are higher than those in younger women, a finding consistent with prior reports.^29^ While our cross‐sectional design does not allow direct measurement of HPV persistence duration, we hypothesize that this association may reflect age‐related immune senescence, which impairs HPV clearance and increases the likelihood of persistent infections.^46^, ^47^ A prior longitudinal study has demonstrated that older women with methylation‐positive results have a significantly higher proportion of progression to severe lesions compared to younger women,^48^ and prolonged HPV persistence correlates with elevated methylation levels.^30^, ^49^ However, the small number of CIN2+ cases and lack of longitudinal data in our cohort limits causal inference, and these hypotheses require validation in prospective screening populations.

This study has several important limitations that warrant explicit consideration. First, although we matched the younger control group by HPV and cytology results, our study lacks systematic data on participants' prior screening histories (e.g., prior HPV or cytology results, screening intervals, prior treatments). The absence of these data can introduce several forms of bias: (1) selection bias—older women referred for colposcopy in our cohort may be enriched for individuals with less frequent previous screening or with delayed care, which could increase the observed prevalence of advanced lesions; (2) information bias—unknown prior test results limit our ability to separate incident from prevalent/persistent disease; and (3) confounding—differences in screening behavior or access to care between age groups may confound the association between age and methylation levels. Consequently, the observed higher proportion of advanced lesions and the apparently superior specificity of methylation testing in the ≥50 group should be interpreted cautiously. Second, the absolute number of CIN2+ cases in the ≥50 group was limited (*n* = 44; CIN2 = 8, CIN3 = 15, SCC = 18, AD = 3), which reduces the precision of sensitivity and specificity estimates and limits generalisability. Wider confidence intervals reflect this limited sample size and underscore the need for replication. To mitigate these concerns, future research should use population‐based or prospective cohorts with systematic capture of prior screening history (or linkage to screening registries), consider matching or stratifying by screening intensity, and aim for larger multi‐center sample sizes to validate the performance estimates observed here.

## 5 CONCLUSIONS

Our study suggests that methylation detection can significantly reduce the risk of missed diagnoses in postmenopausal women. The *PAX1/JAM3* methylation panel could serve as a triage tool for CC among women undergoing evaluation for cervical disease, particularly in postmenopausal patients and may enhance colposcopy by providing more accurate lesion diagnosis.

## AUTHOR CONTRIBUTIONS

**Huanzi Peng:** Writing – original draft; writing – review and editing; conceptualization; data curation; methodology. **Jing Li:** Writing – original draft; validation; formal analysis; project administration; supervision. **Qun Zhou:** Writing – review and editing; conceptualization; investigation; methodology; project administration; visualization. **Hui Zhou:** Visualization; writing – original draft; formal analysis; data curation. **Jiaqi Peng:** Validation; conceptualization; writing – review and editing; data curation; supervision; investigation. **Jing Wang:** Formal analysis; data curation; writing – original draft; validation; software. **Pei Liu:** Conceptualization; supervision; project administration; resources. **Kun He:** Methodology; visualization; validation; formal analysis; project administration. **Wene Liu:** Methodology; validation; project administration; software; supervision. **Ping Tan:** Conceptualization; investigation; visualization; resources. **Lei Li:** Conceptualization; methodology; validation; writing – review and editing. **Xiaobing Xie:** Writing – original draft; writing – review and editing; conceptualization; funding acquisition; supervision; resources.

## FUNDING INFORMATION

This work was supported by Zhongnanshan Medical Foundation of Guangdong Province (ZNSXS‐20240045); Hunan University of Traditional Chinese Medicine's Discipline Construction Project of “Exposing the List and Taking the Lead” (22JBZ037); Hunan University of Chinese Medicine teaching reform key project (2020‐JG002); Open Research Fund Program of the State Key Laboratory of Virology of China (2024KF001); First Class Discipline Open Fund Project of Hunan University of Chinese Medicine (2018YXJS02); Changsha Natural Science Foundation Project (kq2502082); Independent Research Fund of State Key Laboratory of Complex, Severe and Rare Diseases in Peking Union Medical College Hospital (2025‐I‐ZD‐001 and 2025‐O‐ZD‐003); Key Research Project of Beijing Natural Science Foundation (No. Z220013); CAMS Innovation Fund for Medical Sciences (CIFMS) (No. 2024‐I2M‐C&T‐B‐029); National High Level Hospital Clinical Research Funding (2022‐PUMCH‐ B‐083, 2022‐PUMCH‐C‐010, 2022‐PUMCH‐C‐022 and 2022‐PUMCH‐D‐003); Peking Union Medical College Hospital Talent Cultivation Program (Category D) (No. UHB12577); National Key Clinical Specialty Construction Project (U114000); The Beijing Key Clinical Specialty Project (Beijing Chuiyangliu Hospital 2025).

## CONFLICT OF INTEREST STATEMENT

The authors declare that they have no competing interests.

## ETHICS STATEMENT

This study strictly followed institutional and National Research Council ethics guidelines for research involving human subjects, and was approved by the Ethics Committee of The First Hospital of Hunan University of Chinese Medicine (HN‐LL‐LW‐2025‐035). Women signed and gave informed consent to participate in this study.

## Supporting information


**Data S1** Supplementary Materials.

## Data Availability

The data that support the findings of this study are available from the corresponding author upon reasonable request.
