# Supplementary material for: Enhanced diagnostic accuracy of high‐grade cervical intraepithelial neoplasia in postmenopausal women through PAX1 / JAM3 methylation analysis
Source: Int J Cancer. 2025 Nov 14;158(4):1116–25. doi: 10.1002/ijc.70245 (PMC12712368; doi:10.1002/ijc.70245)
Supplement: Supplementary file 1 — Data S1 Supplementary Materials. [file IJC-158-1116-s001.pdf]

Supplementary Materials

Enhanced diagnostic accuracy of high-grade cervical intraepithelial neoplasia in postmenopausal women through *PAX1*/*JAM3* methylation analysis

Huanzi Peng, Jing Li, Qun Zhou, Hui Zhou, Jiaqi Peng, Jing Wang, Pei Liu, Kun He, Wene Liu, Ping Tan, Li Lei, Xiaobing Xie

Table of Contents

Table S1 Significance analysis between sensitivity/specificity of different screening methods 2
Table S2. Clinical performance of different tests in triaging women under 50 years 3
Figure S1 The methylation levels of *PAX1* and *JAM3* in women aged over and under 50 years with different grade of cervical lesions 4

Table S1 Significance analysis between sensitivity/specificity of different screening methods

***P* values**	CISCER	*PAX1*m	*JAM3*m	hrHPV+	HPV 16/18+	LBC ≥ ASC-US
**CIN2+**						
**Sensitivity**						
CISCER	1	1	0.196	1	6.59e-04	0.039
PAX1m	1	1	0.352	0.676	2.17e-03	0.087
JAM3m	0.196	0.352	1	0.089	0.059	0.604
hrHPV+	1	0.676	0.089	1	1.56e-04	0.014
HPV 16/18+	6.59e-04	2.17e-03	0.059	1.56e-04	1	0.252
LBC ≥ ASC-US	0.039	0.087	0.604	0.014	0.252	1
LBC & hrHPV	0.196	0.352	1	0.089	0.059	0.604
**Specificity**						
CISCER	1	0.818	0.199	3.39e-42	2.74e-03	1.93e-17
*PAX1*m	0.818	1	0.414	1.20e-43	6.71e-04	1.36e-18
*JAM3*m	0.199	0.414	1	1.21e-54	8.37e-06	4.89e-24
hrHPV+	3.39e-42	1.20e-43	1.21e-54	1	2.79e-30	1.42e-09
HPV 16/18+	2.74e-03	6.71e-04	8.37e-06	2.79e-30	1	4.41e-09
LBC ≥ ASC-US	1.93e-17	1.36e-18	4.89e-24	1.42e-09	4.41e-09	1
LBC & hrHPV	7.95e-22	4.67e-23	1.93e-29	1.42e-06	1.50e-12	0.235
**CIN3+**						
**Sensitivity**						
CISCER	1	1	0.199	1	2.46e-04	0.014
*PAX1*m	1	1	0.429	1	1.26e-03	0.046
*JAM3*m	0.199	0.429	1	0.429	0.031	0.372
hrHPV+	1	1	0.429	1	1.26e-03	0.046
HPV 16/18+	2.46e-04	1.26e-03	0.031	1.26e-03	1	0.312
LBC ≥ ASC-US	0.014	0.046	0.372	0.046	0.312	1
LBC & hrHPV	0.107	0.26	1	0.26	0.066	0.562
**Specificity**						
CISCER	1	0.853	0.23	2.90e-41	0.011	4.79e-16
*PAX1*m	0.853	1	0.407	1.29e-42	3.78e-03	4.60e-17
*JAM3*m	0.23	0.407	1	3.72e-46	1.31e-04	7.68e-20
hrHPV+	2.90e-41	1.29e-42	3.72e-46	1	1.49e-30	6.54e-10
HPV 16/18+	0.011	3.78e-03	1.31e-04	1.49e-30	1	7.53e-09
LBC ≥ ASC-US	4.79e-16	4.60e-17	7.68e-20	6.54e-10	7.53e-09	1
LBC & hrHPV	2.78e-20	2.20e-21	2.36e-24	6.57e-07	3.23e-12	0.246

CISCER: *PAX1*m/*JAM3*m. *PAX1*m: the methylation of PAX1 gene; *JAM3*m: the methylation of JAM3 gene. hrHPV: HPV 16, 18, 31, 33, 35, 39, 45, 51, 52, 56, 58, 59, 66 and 68 types. HPV-16/18: HPV16 and (or) HPV18 types. LBC (≥ ASC-US): the liquid-based cytology results were defined as cytology were ASC-US or worse. LBC & hrHPV: positive in cases of HPV 16/18 infection, non-16/18 hrHPV infection with cytology ≥ ASC-US, or HPV-negative with cytology ＞ ASC-US.

Abbreviations: CIN, cervical intraepithelial neoplasia; hrHPV, high-risk human papillomavirus; LBC, liquid-based cytology; ASC-US, atypical squamous cells of undetermined significance.

Table S2. Clinical performance of different tests in triaging women under 50 years

	Sensitivity % (95% CI)	Specificity % (95% CI)	PPV % (95% CI)	NPV % (95% CI)	AUC
**CIN2+ lesions**					
PAX1m	60 [46.4-73.6]	94.4 [90.9-98]	76.9 [63.7-90.1]	88.4 [83.7-93.2]	0.772 [0.687-0.858]
JAM3m	52 [38.2-65.8]	98.8 [97.1-100]	92.9 [83.3-102.4]	87 [82.1-91.8]	0.754 [0.676-0.832]
**CISCER**	72 [59.6-84.4]	94.4 [90.9-98]	80 [68.3-91.7]	91.6 [87.4-95.8]	0.832 [0.752-0.912]
**LBC (≥ASC-US)**	80 [68.9-91.1]	57.4 [49.8-65]	36.7 [27.6-45.7]	90.3 [84.6-96]	0.687 [0.594-0.781]
**hrHPV+**	92 [84.5-99.5]	21.6 [15.3-27.9]	26.6 [20-33.2]	89.7 [80.2-99.3]	0.568 [0.499-0.637]
**HPV16/18+**	48 [34.2-61.8]	84 [78.3-89.6]	48 [34.2-61.8]	84 [78.3-89.6]	0.66 [0.562-0.757]
**LBC & hrHPV**	90 [81.7-98.3]	51.2 [43.5-58.9]	36.3 [27.8-44.8]	94.3 [89.5-99.2]	0.706 [0.626-0.786]
**CIN3+ lesions**					
PAX1m	72 [54.4-89.6]	88.8 [84.2-93.3]	46.2 [30.5-61.8]	96 [93-98.9]	0.804 [0.693-0.914]
JAM3m	64 [45.2-82.8]	93.6 [90.1-97.1]	57.1 [38.8-75.5]	95.1 [92-98.2]	0.788 [0.676-0.9]
**CISCER**	84 [69.6-98.4]	87.2 [82.4-92]	46.7 [32.1-61.2]	97.6 [95.3-99.9]	0.856 [0.76-0.952]
**LBC (≥ASC-US)**	80 [64.3-95.7]	52.4 [45.2-59.6]	18.3 [11.1-25.6]	95.1 [91-99.3]	0.662 [0.548-0.776]
**hrHPV+**	96 [88.3-100]	20.3 [14.6-26.1]	13.9 [8.7-19]	97.4 [92.5-102.4]	0.582 [0.514-0.649]
**HPV16/18+**	60 [40.8-79.2]	81.3 [75.7-86.9]	30 [17.3-42.7]	93.8 [90.1-97.5]	0.706 [0.582-0.83]
**LBC & hrHPV**	96 [88.3-100]	46.5 [39.4-53.7]	19.4 [12.4-26.3]	98.9 [96.6-101.1]	0.713 [0.638-0.787]

CISCER: $PAX1^m$/$JAM3^m$. $PAX1^m$: the methylation of PAX1 gene; $JAM3^m$: the methylation of JAM3 gene. hrHPV: HPV 16, 18, 31, 33, 35, 39, 45, 51, 52, 56, 58, 59, 66 and 68 types. HPV-16/18: HPV16 and (or) HPV18 types. LBC (≥ ASC-US): the liquid-based cytology results were defined as cytology were ASC-US or worse. LBC & hrHPV: positive in cases of HPV 16/18 infection, non-16/18 hrHPV infection with cytology ≥ASC-US, or HPV-negative with cytology＞ASC-US.

Abbreviations: CI: confidence interval; PPV: positive predictive value; NPV: negative predictive value; AUC: area under the curve; CIN, cervical intraepithelial neoplasia; hrHPV, high-risk human papillomavirus; LBC, liquid-based cytology; ASC-US, atypical squamous cells of undetermined significance.

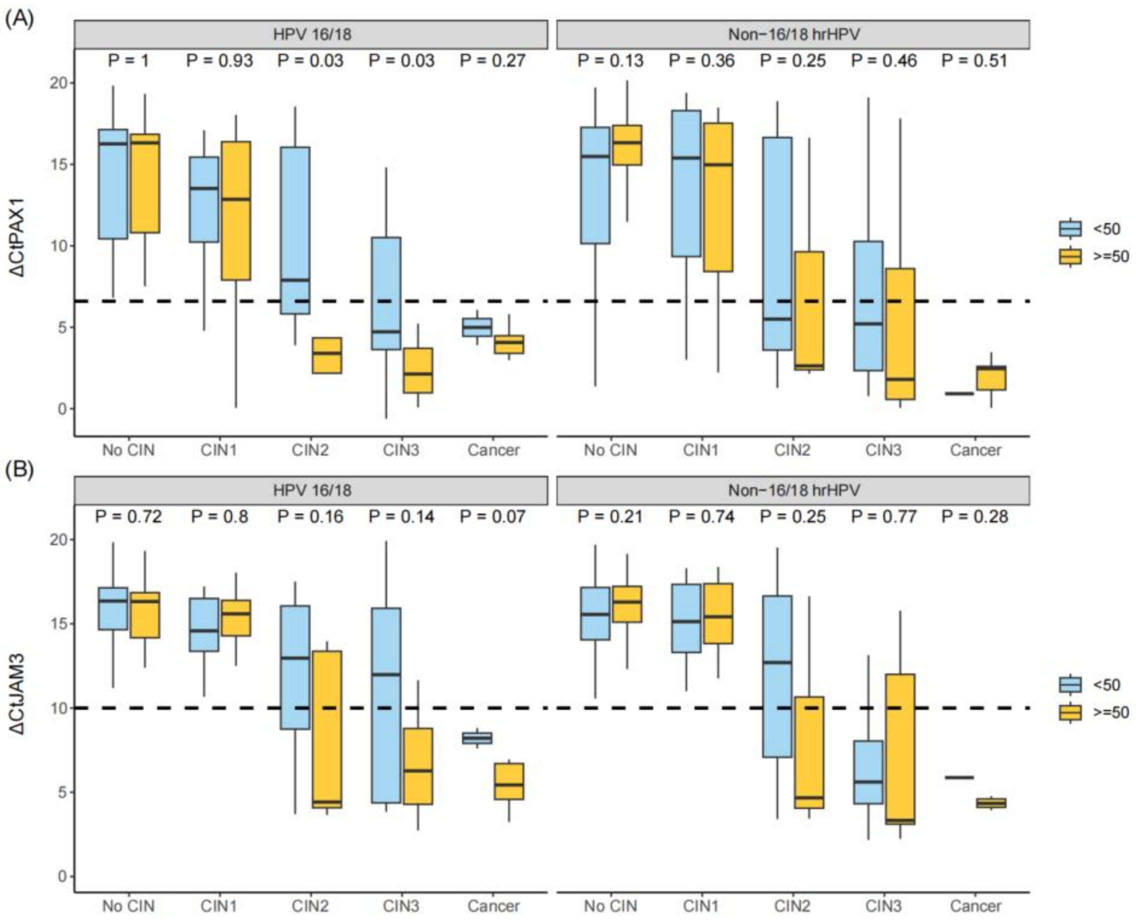

Figure S1 The methylation levels of *PAX1* and *JAM3* in women aged over and under 50 years with different grade of cervical lesions

(A) ΔCt values of *PAX1* gene in patients aged over and under 50 years, infected with HPV 16/18 or non-16/18 hrHPV

(B) ΔCt values of *JAM3* gene in patients aged over and under 50 years, infected with HPV 16/18 or non-16/18 hrHPV
